# MACC1-induced migration in tumors: Current state and perspective

**DOI:** 10.3389/fonc.2023.1165676

**Published:** 2023-03-27

**Authors:** Tim Hohmann, Urszula Hohmann, Faramarz Dehghani

**Affiliations:** Department of Anatomy and Cell Biology, Martin Luther University Halle-Wittenberg, Halle (Saale), Germany

**Keywords:** MACC1, migration, adhesion, cytoskeleton, cancer

## Abstract

Malignant tumors are still a global, heavy health burden. Many tumor types cannot be treated curatively, underlining the need for new treatment targets. In recent years, metastasis associated in colon cancer 1 (MACC1) was identified as a promising biomarker and drug target, as it is promoting tumor migration, initiation, proliferation, and others in a multitude of solid cancers. Here, we will summarize the current knowledge about MACC1-induced tumor cell migration with a special focus on the cytoskeletal and adhesive systems. In addition, a brief overview of several *in vitro* models used for the analysis of cell migration is given. In this context, we will point to issues with the currently most prevalent models used to study MACC1-dependent migration. Lastly, open questions about MACC1-dependent effects on tumor cell migration will be addressed.

## Introduction

Malignant tumors are one of the most common and often deadly health problems throughout the world. Analysis for 2020 estimated 19.3 million new cases of cancer and 10 million deaths from cancer (1). Thus, it is without a doubt still a necessity to better understand the mechanisms of tumor development and expansion and to improve the treatment of cancer. In 2009, the gene metastasis associated in colon cancer 1 (*MACC1*) was discovered in colorectal cancer (2) and connected to increased metastasis, cell survival, proliferation, and migration. Afterward, MACC1 was quickly found to be involved in tumor formation and migration in a multitude of different solid tumors, including glioblastoma, ovarian carcinoma, gastric cancer, hepatocellular carcinoma, and others (3). Notably, in many instances, MACC1 expression was additionally linked to increased metastasis formation, being the main reason for cancer mortality (3). Interestingly, circulating MACC1 transcripts or protein levels can also serve as predictive markers for tumor progression, as demonstrated in patients with colorectal, pancreatic, gastric, lung, and breast cancer (4–9).

Due to its clinical relevance, MACC1-associated signaling was intensively studied. Thus, many upstream and downstream targets of MACC1 were identified and associated with typical tumor features such as NANOG and OCT4 with dedifferentiation, VEGF, and TWIST1/2 with angiogenesis (3, 10, 11). Notably, in almost all studies, MACC1 expression was associated with a more migratory phenotype (2, 3, 12, 13). Attainment of a migratory phenotype is a key feature in the metastatic cascade and a highly complex phenomenon (14). Cell migration in complex environments necessitates cells to generate propulsive forces, change shapes, form (transient) adhesions with the extracellular matrix (ECM) and neighboring cells, and remodel the extracellular space (15, 16). Consequently, the migratory process is not only highly important during tumor spreading but also involves a multitude of complex processes.

Thus, this review aims to summarize the different downstream effects of MACC1 on tumor migration and how these can be integrated into the cytoskeletal and adhesive systems. Furthermore, we want to point out open questions and additional approaches to elucidate MACC1-dependent effects on cellular migration further.

## The cytoskeleton, adhesion, and cell migration

The migration of cells is an essential part of cancer metastasis, depending on the reorganization of the cytoskeleton, cell–cell, and cell–matrix adhesions. Here, only a brief review of the cytoskeleton, adhesion, and its relation to migratory processes is given. For further information, the interested reader is referred to the following reviews (17–31).

### The cytoskeleton

The cytoskeleton is a network consisting of actin filaments, microtubules, and intermediate filaments. The (sub-) structures formed by each of those elements are not independent, but coupled, increasing the complexity of the system (17). For this review, we will focus on microtubules and actin due to their crucial role in cell migration and on vimentin as it was often found to be regulated by MACC1 (32–35).

#### Actin

Actin filaments can form different types of cytoplasmatic structures, such as the actin cortex, the dendritic actin network forming the lamellipodium, and stress fibers.

The lamellipodium is a flat, dendritic actin structure associated with cellular movement in 2D and 3D environments, yet its occurrence in 3D depends on the physical properties of the surrounding cells (36). Actin in the lamellipodium is nucleated by the Arp2/3 complex, quickly creating large protrusions that extend the cells’ exterior by pushing forces created during actin polymerization (37). Arp2/3 is activated *via* the Scar/WAVE complex which in turn is regulated by the small RhoGTPase Rac1 (38). In a 3D environment, N-WASP was shown to induce ARP2/3 activation, mostly independent of Rac1 (39, 40). In addition to these proteins, others such as anti-capping proteins like Ena/vasodilator-stimulated phosphoprotein (VASP), capping proteins, cross-linkers, and polymerization-limiting factors like arpin are necessary to regulate the formation of the lamellipodium (17). During the expansion of the lamellipodium, new cell–substrate adhesions are formed, increasing the lifetime of the lamellipodium, responsible for a movement in the direction of high cell–ECM adhesiveness (41, 42).

In contrast to the lamellipodium, stress fibers are formed by bundles of parallel or anti-parallel-oriented actin filaments (43). Stress fibers are bundled by cross-linkers such as α-actinin, fascin, and filamin and connected to focal adhesions (17). Anti-parallel stress fibers can additionally contain myosin and, thus, generate contractile forces (44). Given that contractility is regulated by myosin, stress fiber contractility is often regulated similarly, *via*, e.g., phosphorylation of the regulatory light chain and activation of the myosin light chain kinase either *via* the small GTPase RhoA or Ca^2+^, respectively (45). The formation of stress fibers depends on the activation of the formin mDia1 and RhoA (46, 47). Stress fibers do not form protrusions but are thought to generate contractile forces to retract the rear, modify the ECM *via* generated tension, or transmit traction forces to the substrate for effective cell body translocation (48–50).

The last structure described here is the actin cortex, forming a contractile structure located beneath the plasma membrane. It consists of actin filament bundles, cross-linkers (α-actinin, fascin, etc.), myosin, actin polymerization factors (ARP2/3, mDia1), ERM family members (ezrin, radixin, moesin), and others (51, 52). In terms of migration, the so-called blebbing needs to be mentioned. Blebs are protrusions formed in regions where the actin cortex locally ruptures or is detached from the membrane, so that hydrostatic pressure inside the cell causes small cell protrusions (blebs) (17). Of note, this mode of migration is suitable for migration in low adhesive environments (53–56). Another property of the cortex is its tension, determining cell shape and thus migration regulated by activation of myosin and actin polymerization (57–59).

#### Microtubules

Microtubules are hollow filaments consisting of α- and β-tubulin. Due to their larger size and organization, microtubules can withstand larger compressive forces than actin and intermediate filaments (60). Thus, microtubules serve as tracks for intracellular transport. Except for some cases, microtubules are mostly regarded as indirect promoters of migration, independent of their mechanical contribution (61–63). As microtubules are polarized, molecular motors can transport cargo directionally along formed tracks. The cargo can contain lipids to increase the surface area for protrusions, secretory proteins, integrins, small GTPases (Rac, CDC42), proteases, etc. (17, 27), all associated with cell migration. Furthermore, mRNA for leading edge components, such as the ARP2/3 complex, profilin, or β-actin, is transported along microtubule tracks (64–66).

Notably, the actin cytoskeleton and microtubules are inherently coupled, due to a multitude of microtubule regulators, such as APC or mDia1, that bind and regulate actin and due to actin–microtubule cross-linking factors such as MACF1 (17, 27). Furthermore, (de-)polymerization of microtubules could be linked to Rac1 or RhoA signaling, respectively, *via* microtubule-regulated guanine exchange factors (GEFs) (27). Consequently, changes in the regulation of one cytoskeletal network can affect the other directly or indirectly.

#### Vimentin

Vimentin belongs to the class of type III intermediate filaments, forming homopolymers of vimentin monomers, and it is expressed in most cancer and precursor cells. Vimentin plays an important role in migration. Its upregulation correlates with the epithelial–mesenchymal transition (EMT), associated with metastasis (67). Consequently, motile and invasive cells show higher vimentin expression, and the knockdown of vimentin impairs migration (68). One mechanism of vimentin action is its function as a guiding structure for microtubule growth, necessary for maintaining cell polarity (69). Despite microtubule-associated effects, vimentin co-regulates the organization of the actin cytoskeleton. Thus, vimentin can either directly bind to actin (70) or indirectly *via*, e.g., plectin (71). In addition, vimentin depletion caused the induction of RhoA and myosin activity and, consequently, stress fiber assembly (72, 73). In astrocytes, vimentin was found to be necessary to maintain cell polarization of leader cells in wound-healing assays *via* control of forces, attributed to a lower degree of focal adhesion concentration at the cell front (74).

Furthermore, vimentin is involved in nuclear positioning, a key element in cell migration, as the nucleus is the largest and stiffest organelle of the cell (75, 76). Likewise, vimentin supports the cell against compressive stress as experienced during tumor growth, promoting cell migration and invasion (77, 78). In line with this idea, vimentin regulates migration in dense but not in sparse cultures, by induction of a stiffer, less deformable phenotype (79).

### Cell adhesions

Cells need anchorage points for migration allowing them to transmit forces. The most common forms are specific cell–cell adhesions and cell–matrix adhesions. Because of the highly complex nature of both adhesive systems, we will focus on integrin-based cell–matrix adhesions and cadherin-mediated cell–cell adhesions.

#### Cell–matrix adhesions

Cell–matrix adhesions—as referred to here—are considered connections of transmembranous integrins and extracellular matrix components such as collagen, fibronectin, and laminin. These bindings result in the formation of adhesion complexes, connecting the ECM to the actin cytoskeleton. The best-characterized cell–matrix adhesion type is the focal adhesion, containing among others integrins, paxilin, focal adhesion kinase (FAK), talin, vinculin, actin, and actin-regulating proteins (80–83). Notably, these molecules are also associated with cell signaling (especially FAK) and mechanotransduction (talin, vinculin), respectively. The full consensus integrin adhesome contains more than 60 components (84). Integrin-mediated cell–matrix adhesions sense and transmit biochemical signals about the ECM composition and mechanics into the cell interior. Thus, they are responsible for directed cell migration in the direction of more rigid substrates (durotaxis), along chemical gradients (chemotaxis), and in the direction of higher ECM concentration (haptotaxis) (85). Signals are transmitted to the cell interior *via* activation of, e.g., YAP/TAZ or SRF (86). Further signaling molecules associated with cell–matrix adhesions are FAK, Src, paxillin, and others, all associated with cell migration (24). FAK signaling can promote cell migration *via* Rac1-induced actin polymerization, using different routes through either PI3K or p130cas/Crk/DOCK180 signaling (87). Furthermore, FAK can suppress stress fiber formation *via* RhoA inhibition and regulate several GTPase-activating proteins (GAP) and activate N-WASP to facilitate Arp2/3 activation at the leading edge (87).

As cell–matrix adhesions are directly connected to the actin cytoskeleton, it is not surprising that a multitude of interactions between focal adhesions and microtubules and vimentin exist. For example, it was demonstrated that microtubules can target focal adhesions, especially at the rear of the cell, fastening their dissociation (27). On the other hand, microtubules support the formation of focal adhesions at the front *via* the transport of integrins (88, 89). Similarly, vimentin localizes at focal adhesions (90), directly interacting with integrin subunits (91, 92), and incorporates into forming (nascent) and mature adhesions (93). In addition, vimentin was found to be involved in integrin trafficking to the leading edge (94).

#### Cell–cell adhesions

Cell–cell adhesions couple neighboring cells, not only functioning as a signaling hub but also introducing mechanical coupling involved in collective cell migration. The most studied class of cell–cell adhesion molecules is cadherins. They are calcium-dependent transmembrane proteins, consisting of multiple members, including N-cadherin, E-cadherin, and P-cadherin, and form homotypic and heterotypic adhesive bonds. Despite cadherins, adhesion complexes contain β-catenin, α-catenin, p120 catenin, vinculin, GEFs, VASP, and others and are connected to the actin cytoskeleton (22, 95). Of note, GEFs, VASP, and others are related to actin remodeling (95). Thus, classical cadherins mechanically connect the actin cytoskeleton of neighboring cells, and therefore, the tension on the actin cytoskeleton can be transferred across multiple cells. The transferred tension can in turn stabilize the adhesion (96–98), resulting in cortical stiffening (98). Cadherin binding also regulates Rac1 and Arp2/3, inhibiting protrusion formation in follower cells during collective migration (99), probably partly dependent on its impact on cortex organization. Cadherin adhesions further alter actin cytoskeleton organization and promote tension by activation of RhoA and Cdc42 (100, 101). Additionally, the application of tension on E-cadherin activates PI3K in EGFR dependence, resulting in integrin-dependent cell–matrix adhesions and ROCK-induced contractility (102). Similarly, P-cadherin was found to promote focal contact formation (103). It is also noteworthy that cadherin types have different functions and associations with (collective) cell migration. E-cadherin and N-cadherin, for example, are involved in EMT and associated with a more (N-cadherin) or less (E-cadherin) migratory phenotype (104). Yet, the exact role of E-cadherin and cell–cell adhesion in cell migration, in general, is still a matter of debate, as E-cadherin was found in migrating tumor cells and collective migration was the dominant form of migration in tumors (21, 105, 106). Similarly, for E-cadherin and P-cadherin, different roles in force transmission were found. While E-cadherin strengthens cell–cell adhesions, P-cadherins regulate the tension a focal adhesion can transmit, but P-cadherin can partially substitute E-cadherin in case of E-cadherin loss (107).

Given the close connection between adherence junctions and the actin cytoskeleton, numerous interactions between cell–matrix interactions, intermediate filaments, and microtubules were found. For example, a formed keratin–cadherin complex was demonstrated to be involved in directional migration (108), while microtubules are involved in the transport of N-cadherin and p120 catenin to adherens junctions (109, 110). Cell–ECM adhesions to fibronectin were found to inhibit the formation of E-cadherin-dependent cell–cell junctions (111). Similarly, β1 integrin binding triggered the scattering and disassembly of cell–cell contacts (112). One explanation may be the binding-induced outside-in signaling of integrins, *via* FAK and Src that can destabilize cell–cell adhesions (23).

## Models to study cell migration

**Figure 1:**
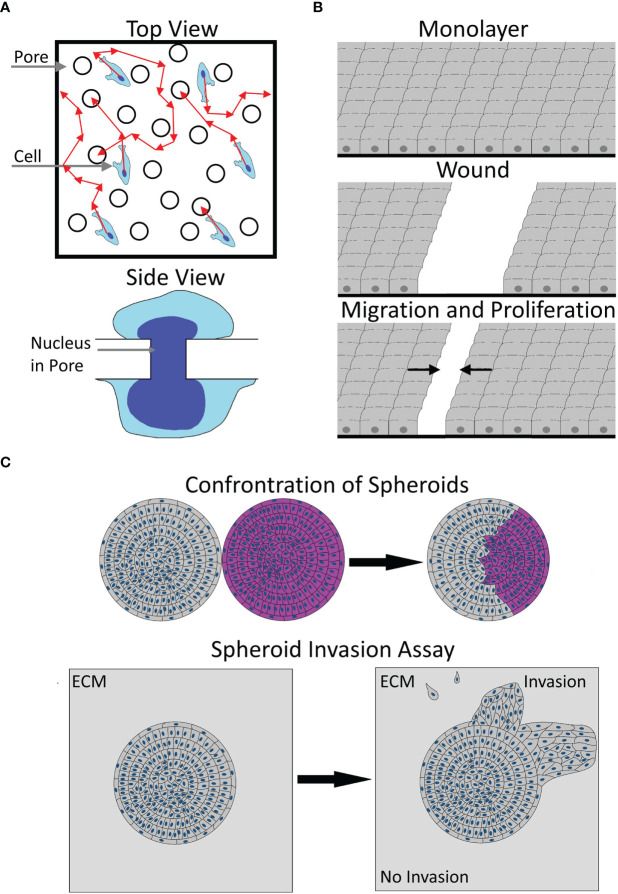
Illustration of migration models. **(A)** A sketch of the transwell model from the top (top) or side view (bottom). Please note the uneven distribution of pores and unclear cell path (red arrows) before contacting a pore in the top view. The side view shows the necessary, potentially rate-limiting deformation of the nucleus to pass a pore. **(B)** The working principle of a scratch wound assay, starting from an unscratched monolayer, via wounding, to subsequent wound closure by proliferation and migration. Arrows denote the average direction of migration. **(C)** Two types of 3D migration models. The top image shows a typical spheroid confrontation assay, bringing the spheroids of two cell populations (gray and pink) into contact, with the subsequent fusion of spheroids and intraspheroidal migration. The bottom image illustrates a typical spheroid invasion assay, showing an embedded spheroid, showing collective and single-cell invasion into the ECM. Arrows illustrate the transition from the start to the end of the experiment.

Given the complexity and entanglement of the cytoskeletal and adhesive systems, the choice of the model system and evaluation parameters is highly important to differentiate between different types of effects involved in migration, e.g., effects on cell polarization, cooperativity, and chemotaxis. Thus, we briefly summarize the most popular *in vitro* migration models and address their advantages and disadvantages. Therefore, we will group the models according to the dimensionality of the system as either 1D, 2D, or 3D and as endpoint or dynamic measurements. A brief summary of the presented models is given in **Table 1**, and some models are illustrated in **Figure 1**.

**Table 1:** Summary of the migration models.

Model	Static	Dynamic	Advantages	Disadvantages
1D migration	x	x	- Stable gradients - Tunable surfaces (ECM, mechanics) - Single- and collective cell migration	- Complex setups - Potentially complex data analysis
2D single cells		x	- Simple setup - Tunable surfaces (ECM, mechanics) - Low cell numbers needed - Well plate compatible	- No cell–cell interactions - Potentially complex analysis - Unphysiological surroundings - No chemotaxis
Scratch assay	x	x	- Well plate compatible - Directional cell movement - Tunable surfaces (ECM)	- Variability of manual scratches - Can damage coatings - Released factors of damaged cells - No chemotaxis - No differentiation between migration, viability, and proliferation as an endpoint assay
Transwell	x		- Easy implementation - Low cost - High throughput - Easy analysis - Chemotactic gradients	- Only single-cell properties - High amount of preliminary experiments needed - Unclear interpretation - Many experimental uncertainties - Unphysiological surfaces - Unstable, non-linear gradients
Spheroid migration assay	x	x	- Most physiological conditions - Tunable surroundings (ECM, mechanics, stroma cells) - 2D and 3D migration possible - Single- and collective cell migration	- Complex setup - Complex analysis - Not suitable for all cell types

x denotes if the migration model is usually performed as static or dynamic experiment.

1D migration can be realized either *via* thin strips of adhesive substrate surrounded by non-adhesive substrate or *via* narrow channels that cells can migrate through (113–118). Such models are considered to represent migration along fibers in confinement (119) and are appropriate to either analyze single-cell migration or collective migration of groups of cells in a continuous manner (114, 116). Furthermore, some systems, especially microfluidic ones, can be used to induce long-term stable chemotactic gradients (120). Consequently, 1D systems are suitable to assess chemotaxis, but also migration under confinement. On the other hand, such setups might be complex to implement and analyze. Such approaches might be useful to elucidate the effects of MACC1 on chemotaxis and to decouple the effects on random migration from chemotaxis. Currently, the most prominently used Boyden chamber assays cannot distinguish well between both. Please see below for more details.

Looking at 2D systems, three common models will be discussed: sparsely seeded cells on a 2D substrate, the scratch wound, and the transwell or Boyden chamber assay. While technically not entirely 2D, the dimensionality of transwell assays is closest to a 2D system, rather than 1D or 3D.

The simplest form to analyze migration in 2D is to sparsely seed cells and monitor the movement of individual cells *via* time-lapse microscopy. Typically, cell speed and the sense of directionality of cells are measured (13, 121, 122). The conduction of such experiments is comparably simple, and surfaces can be modified both in terms of functionalization, *via*, e.g., ECM components and stiffness (117). Yet, analysis can be more complex. If done manually, the throughput is limited and results may vary between different raters. Automatic approaches allow for a high throughput (121, 123, 124) but are normally optimized to a certain cell appearance and morphology. Thus, they either need significant parameter tuning or might even be unusable for certain cell types, albeit machine learning systems help to overcome this issue (123, 124). As only single cells are analyzed, cell–cell interactions are neglected, but the effects of substrates, interventions and environmental stiffness on cytoskeletal dynamics can be analyzed. Notably, for MACC1, such analysis is mostly missing.

When using the scratch wound assay, cells are seeded to form a dense monolayer, and afterward, an artificial wound is created (125, 126). Notably, the surfaces can be functionalized using ECM components. Afterward, the wound closure is monitored for several days, either as endpoint measurement or in a continuous manner. Typically, the rate of wound closure is used as a proxy for migration (127, 128), albeit a more complex analysis can be performed to obtain additional information (129–132). In the simplest form, conducted as an endpoint assay without complex surface functionalization, the scratch assay is comparably simple to perform and analyze. Yet, the scratch assay has several disadvantages, such as the scratch procedure itself suffers—if done manually—from a large variability (133, 134) and is inducing significant damage to the remaining cells, *via* factors released from damaged cells (134). Furthermore, the scratch procedure may damage surface modifications (133, 135). To circumvent the abovementioned issues, the cell exclusion assay can be used. In principle, it is identical to the scratch assay except that cells are seeded into a culture vessel containing a block of defined size so that cells cannot enter this area. For the experiment, the block is removed and migration is monitored as mentioned before, assuring that no cell death occurs. Yet, precaution must be taken that none of the cells crawls under the used block (136). Independent of the usage of the scratch or exclusion assay, cell proliferation has to be taken into consideration, as most of these assays are performed for at least 1 day, so the final readout will be a mixture of proliferation and migration (137). While attempts are being made to detect cell divisions in parallel in phase contrast images (138–141), the decoupling of proliferation and migration in these assays remains an issue. Furthermore, if interventions affect cell viability, they cannot be distinguished from migration using the scratch or cell exclusion assay with wound width as a readout (127). While scratch wound assays were used frequently, when analyzing MACC1-dependent migration, they were most often performed as endpoint assays, unable to differentiate between proliferation and migration. The use of more advanced analysis schemes combined with live-cell imaging (129–132) may help to reveal more details regarding MACC1 effects on (collective) cell dynamics, cell polarization, leader cell determination, etc. Furthermore, by simultaneous detection of proliferation events or *via* inhibition of proliferation (138–142), MACC1-induced effects on proliferation could potentially be decoupled from migration.

The last 2D system discussed here is the transwell or Boyden chamber assay. For the transwell assay, cells are seeded on top of a coated or uncoated porous membrane with a defined pore size, while the culture medium is placed on top and below the cells. Due to this setup, a chemotactic gradient can be generated *via* the addition of a chemoattractant or repellent to the lower or upper compartment, respectively. As readout for migration, the number of cells migrating from the top to the bottom side of the membrane, the number of cells on the bottom of the lower well, or the sum of both is counted. Normally, cell counting is done manually and only at one defined time point. Therefore, the setup and data analysis can be considered rather simple and quick. Of note, there are modified versions of the Boyden chamber assay that do not need manual counting and allow for a continuous data assessment, e.g., the IncuCyte or xCELLigence systems (12, 143, 144). Yet, for MACC1, the standard Boyden chamber assay was the most used one (see **Table 2**). On the other hand, the transwell assay has some severe drawbacks: The size of the pores of the membrane has to be chosen carefully, to fit the overall size of the cell and nucleus, as the nucleus and its deformability are often rate-limiting for cell migration (146, 147). Too large pores would in contrast lead to the unspecific dropping of cells through the membrane (136). The gradient generated is neither constant over time nor linear (148, 149). The generated gradients can nearly vanish after as short as 6 h and degrade to 50% of their initial value after ≈1 min (148). Notably, gradient steepness affects chemotactic response (150–152). When seeding cells, care must be taken to obtain a single-cell suspension, as cell aggregates are slower or even unable to migrate through the membrane pores. For obtaining statistically robust results, it is necessary to have a large number of migrating cells, and as most cell types have different migratory capacities, the experimental endpoint needs to be determined for every single-cell type individually (135). Another issue with the transwell assay is the distribution and size of pores on the membrane, which is not even (136, 153), and thus, the amount of random 2D motion of cells on top of the membrane before reaching a pore is undefined and creates additional measurement uncertainty. A last point to be taken into account is the time between seeding and stopping of the experiment. For times above 24 h, proliferation affects the readout, *via* an increase of cells on the lower membrane and the bottom part of the assay. Thus, for sufficiently long experimental times, the effects of interventions on proliferation cannot be distinguished from migration. Please note that for most migration experiments associated with MACC1, incubation times of 24-48 h were used. Taken together, using the transwell assay, it can be—dependent on the exact setup—highly difficult to elucidate the actual reason for the change of migratory capacity.

**Table 2:** The number and type of *in vitro* assays used to analyze MACC1-dependent migratory effects.

Assay	Endpoint	Dynamic	Sum
1D assays	0	0	0
2D single cells	0	2	2
Scratch	30	3	33
Transwell	69	3^a^	72
3D assays	0	0	0
Others	2^b^	2^c^	4
Sum	101	9	111
Number of studies^d^			75

a Basti et al. and Treese et al. (143, 144) used a modified transwell with the IncuCyte system. Hagemann et al. (12) used a modified transwell with the xCELLigence system.

b Li et al. (145) used a 2D microfluidic migration device. Hagemann et al. (12) used an *ex vivo* OHSC invasion model.

c Hohmann et al. (142) analyzed collective migration in dense monolayers and small cell colonies.

d The overall sum of studies does not match the assay number as some studies employed multiple assays.

For 3D migration models, we limit the description to the spheroid migration/invasion assay. Spheroids are multicellular, spherical objects of one or more cell types, cultured in a low to non-adhesive environment or generated *via* confinement (154), favoring the formation of cell–cell adhesions instead of cell–matrix adhesions. When reaching a critical diameter of 200-500 µm, spheroids develop oxygen, nutrient, and catabolite gradients and, when growing larger, show necrotic cores, recapitulating several key factors of *in vivo* tumors (155). For migration/invasion assays, spheroids can be placed on top of a coated surface or embedded in a hydrogel, mimicking the ECM (155). Independent of the exact experimental settings, spheroids can be imaged continuously or at the beginning and end of the experiment only. A typical readout of such migration experiments is the increase in cell-covered area or tracking of individual migrating cells, to measure speed, morphology, or even migration of cells inside the spheroid (156–160). While such a model represents a more physiological approach, it is typically more time-consuming. Notably, the size of the spheroid needs to be controlled tightly, as this affects its composition and thus the gradients inside the spheroid (155). If spheroids are embedded into hydrogels, the stiffness, pore size, and composition of the hydrogel need to be precisely chosen and reproduced, as all affect cell migration (146, 147, 161, 162). Furthermore, not all cell types and lines form spheroids in all assays. Additionally, if live-cell imaging is performed, together with the analysis of the motion of single cells, the analysis might become highly complex (156, 160).

As a special case, a spheroid confrontation assay can be done. There, cells of one spheroid can migrate into another spheroid of the same or different cell type (155, 159, 160). In principle, similar parameters can be assessed as described before: the infiltration of one spheroid into the other, either as a bulk measurement or on a single-cell basis, and the time of complete fusion (159, 160). All parameters can be considered proxies of cell migration and/or invasion. Usage of the mentioned 3D assays could help to analyze the role of homo- or heterotypical cell–cell interactions during migration in more physiological environments, in terms of dimensionality, stiffness, and chemical composition. Notably, the effect of confinement—drastically altering the motile machinery (163)—can be studied as well. Currently, studies on MACC1 focused on 2D models. Yet, in 2D, cells tend to show drastically different behavior and organization compared with 3D, including morphology, proliferation, cell interactions, and gene expression patterns (161). Thus, the usage of more complex 3D models is expected to yield a rich set of information on MACC1-dependent migration.

## MACC1 in tumor migration

**Figure 2:**
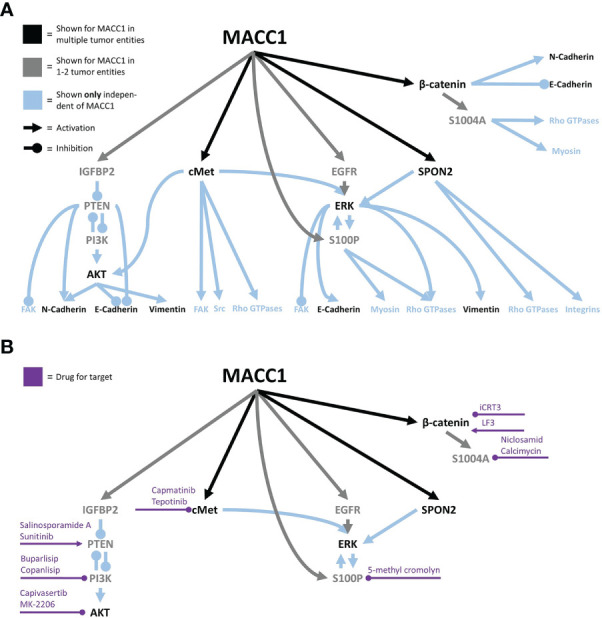
The MACC1 signaling network associated with cell migration **(A)** and potential intervention points, with the associated drugs **(B)**.

Starting with the initial discovery of MACC1, effects on migration were reported (2). Notably, *in vivo* MACC1 was also enriched in tumor buds and cells at the invasive front of colon carcinoma (164, 165), making it tempting to speculate about the role of MACC1 in leader cell determination during collective invasion. Initially, MACC1-induced effects involved the activation of HGF/cMet signaling (2). The following studies supported these findings (12, 33, 166–170). cMet signaling can induce activation of the Src family members, FAK, small Rho GTPases such as Cdc42 or Rac1, and others, all implied in cell migration (171, 172). A pan-cancer database analysis of MACC1 in 33 cancer types generated a consensus list of 1,896 genes correlating with MACC1 in at least half of the tumor entities. The authors found an enrichment of the consensus list in genes associated with cell junction organization, cell–cell junctions, and regulation of cell adhesion and cell junctions, pointing toward a far larger set of MACC1-associated pathways than HGF/cMet (173). A sketch of the current migration-associated network is shown in **Figure 2A**, and the potential intervention strategies are shown in **Figure 2B**. Of note, cMet signaling can induce AKT and ERK signaling (171, 172, 174), both also induced by MACC1 (see below).

Furthermore, MACC1 expression correlated with intracellular and secreted IGFBP2 (175) that was independently found to be collected by integrins causing inhibition of the tumor suppressor PTEN (176, 177). Increased PTEN expression was found after MACC1 silencing in esophageal carcinoma (178), and in hepatocellular carcinoma, MACC1 induced PI3K activation (179). In line with this, several studies reported associations of MACC1 with AKT activation (33, 143, 166, 178–183), suggesting the following MACC1 signaling: MACC1/IGFBP2/PTEN/PI3K/AKT (175, 178, 179, 184). Yet, MACC1 may affect PI3K/AKT signaling also *via* direct interactions with YWHAE (14-3-3 epsilon), as confirmed by affinity capture-MS (185). YWHAE was previously found to induce PI3K and AKT activation (186). Additionally, PTEN was reported to suppress cell–matrix adhesion-associated tumor cell migration by inhibition of FAK (187) and further induce N-cadherin and suppress E-cadherin (188), while PI3K/Akt signaling had the opposite effects (189). Interestingly, MACC1 was shown to interact with MARK2 (Microtubule Affinity Regulating Kinase 2) (185), a protein that induced FAK activation, contractility, and stress fiber formation and was involved in cell polarization (190, 191). Another hint suggesting that MACC1 interacts with cell–cell and cell–matrix adhesions was implied by the interaction of MACC1 with SPON2 in colorectal cancer (192). SPON2 is a known ligand for integrins α4 and β2 (193), linked to integrin α5 and β1 signaling (194), and demonstrated to regulate the expression of Rac1 and Rac2 in dendritic cells (195), resulting in the promotion of lamellipodium formation (196). In contrast, no MACC1-dependent change in the distribution of integrins α5 and β1 was found in glioblastoma cells (13). Furthermore, SPON2 was shown to inhibit E-cadherin and N-cadherin in gastric cancer (197). In addition, MACC1 negatively correlated with the expression of α-actinin (34), a cross-linker necessary for the formation and stability of stress fibers (198). Furthermore, a study in ovarian cancer demonstrated lower cell–matrix adhesion to matrigel after MACC1 silencing in a 2-h time frame covering the onset of cell attachment (199). In line with this, in non-small cell lung and gastric cancer, MACC1 correlated with a lower expression of collagen I and fibronectin (34, 167). Yet, two studies in gastric cancer associated MACC1 with higher fibronectin expression (35, 200). Similarly, adhesion to fibronectin and laminin of glioblastoma cells was not altered after MACC1 overexpression for short interaction times of 1 min (13). In summary, there are clear hints on the effects of MACC1 acting on cell–matrix adhesions, but the effects may depend on the tumor type, analyzed integrins, or the ECM component, and consequently, this aspect needs additional research for a clear picture.

Another signaling route affected by MACC1 is the ERK pathway, as demonstrated in multiple tumor entities (168, 169, 180, 182, 183, 201–203). Potentially, ERK activation is induced by MACC1 *via* sustained EGFR signaling, independent of EGFR expression (201). Moreover, it was demonstrated that the MACC1 target SPON2 can also induce ERK activation in gastric cancer cells (197). Interestingly, ERK1/2 can regulate and is regulated by S100P, which is also induced by MACC1 (204, 205). Previously, S100P was shown to affect myosin II, reduce the number of focal adhesions (206), interact with Cdc42 and Rac1 regulators, and affect cell migration *via* ezrin binding (207). In addition, ERK co-regulates E-cadherin (208) and was shown to be involved in protrusion formation *via* induction of actin polymerization at the leading edge (209). ERK was also suspected to be involved in FAK inactivation, regulation of RhoA and myosin II, downregulation of E-cadherin, and upregulation of N-cadherin and vimentin (197, 209, 210).

Looking further downstream, MACC1 was frequently found to induce a mesenchymal phenotype in multiple tumor types, through the measurement of EMT markers, such as increased expression of vimentin and N-cadherin and reduced expression of E-cadherin (32–34, 166, 169, 170, 182, 203, 211–213). Yet, the intracellular organization of the vimentin cytoskeleton does not appear to be altered in glioblastoma cells upon MACC1 overexpression (13). Direct interactions between E-cadherin and MACC1 were described earlier, being another potential way of E-cadherin regulation *via* MACC1 (214). As discussed before, these molecules are involved in single-cell and collective migration, due to associations with cytoskeletal reorganization and cell–cell coupling. Likewise, MACC1 induced β-catenin expression and phosphorylation, an important signaling and adaptor protein in cell–cell junctions (169, 180, 203, 215–217), negatively regulating E-cadherin (208). As mentioned earlier, MACC1 interacts with YWHAE which was shown to induce lower E-cadherin but higher N-cadherin and vimentin expression (186), being another explanation for the observed effects. Arguments for MACC1 acting on cell–cell adhesions and cytoskeletal organization are supported by a study in HeLa cells showing lowered actin staining upon MACC1 silencing (218) and by experiments on glioblastoma cells showing lower equilibrium cell–cell adhesion after MACC1 overexpression (13). Additionally, α-smooth muscle actin (αSMA) expression, normally expressed in smooth muscles and myofibroblasts, was also positively correlated with MACC1 expression (212). In glioblastoma, MACC1 promoted random motion in 2D, caused by a lower cortical stiffness and accumulation of protrusive actin near the protruding edge (13). Yet, the stiffer and faster random migratory phenotype appears to be tumor type-dependent, as it was not found in colorectal cancer cells (142).

Interestingly, MACC1 also was involved in cytoskeletal organization under metabolic stress in gastric cancer (219). Upon glucose deprivation, gastric cancer cells showed increased formation of stress fibers, caused by DLC3 downregulation and subsequent MACC1 upregulation. Upon silencing MACC1, the induction of stress fibers under metabolic stress was abrogated (219). The same study suggested a MACC1-promoted chemotactic migration along glucose gradients (219). In line with this, MACC1 expression resulted in increased glucose uptake, ATP levels, and lactate production in gastric cancer cells (211). Interestingly, the MACC1 stabilizing long non-coding RNA MACC1-AS is upregulated upon glucose deprivation, further promoting MACC1-induced glycolysis and antioxidant production under metabolic stress (220). Given the fact that the metabolic state and nutrient availability of cells have a large impact on cytoskeletal organization and migration (26, 221), MACC1-induced metabolic adaptation might be another part of the MACC1-regulated migration *in vivo*.

MACC1 expression not only had intracellular effects but also changed the secretion profile of cells. In colorectal cancer, MACC1 expression was connected to increased S100A4 secretion, induced by the β-catenin/TCF4 axis (216). S1004A was previously shown to regulate non-muscle myosin heavy chain and RhoA, leading to chemotaxis and stress fiber formation (222). Additionally, direct interactions with the actin cytoskeleton were found (223). Of note, extracellular S1004A was demonstrated to induce matrix metalloproteinase (MMP) expression of MMP1, MMP3, MMP9, and MMP13, facilitating the degradation and remodeling of the extracellular matrix and thus its migration (223). In agreement, MACC1 was associated with increased expression of several MMPs, namely MMP2, MMP3, and MMP9 (34, 199, 224–226).

Another study performed in colorectal cancer found MACC1 to affect collective but not single-cell migration (142). Interestingly, MACC1-induced effects were fully abrogated when proliferation was inhibited (142). This study points toward another—potentially very important—aspect of MACC1-induced migration that was previously identified independently on MACC1. Proliferation events can cause both a local and even global fluidization of cell layers and thus permit the reorganization and migration of cells (227–232). In light of these observations, the following questions arise: what part of MACC1 promigratory effects is caused by the classical way *via* the cytoskeletal and adhesive dynamics, and what proportion is caused by MACC1-induced increased proliferation? These questions become even more relevant, given the large number of assays used to analyze MACC1-dependent migration that cannot distinguish between proliferation and migration, e.g., scratch assays performed as endpoint assays and long-lasting transwell assays (see **Table 2**).

Inhibiting cancer cell migration and thus ultimately metastasis formation is one approach to fight against cancer. Yet, many clinical trials targeting migration-associated molecules had only limited success, if at all (233, 234). The MACC1-dependent signaling network could be an additional piece to the puzzle helping to bridge the gap between preclinical research and the successful clinical application of anti-migratory drugs. For many MACC1 targets, drugs that are or were in clinical use exist (235–238), albeit for some, available drugs are sparse (see **Figure 2B**). Additionally, some of them, e.g., 5-methyl cromolyn, were not yet clinically tested to the authors’ knowledge and are generally only poorly investigated in human systems (239). Furthermore, lovastatin and rottlerin were shown to transcriptionally inhibit MACC1, but both have diverse off-targets and are only a little specific (128). Consequently, further research into MACC1-specific drugs may be an additional route to inhibit cancer cell migration.

## Conclusion and outlook

In the few years since its discovery, MACC1 has been demonstrated to be a very promising predictive biomarker in a multitude of tumor entities because it induces migration and proliferation among others. On a mechanistic level, effects were mediated very frequently *via* ubiquitous, major pathways, such as cMet, AKT, or ERK. While several studies elucidated further the downstream effects, the current data and understanding of how MACC1-induced effects are transduced to cytoskeletal or adhesive remodeling in detail remain largely elusive. Further research may help to complete this picture and identify potential targets for treatment. As MACC1 is involved not only in cell migration but also in proliferation, functional assays need to take the pro-proliferative effect of MACC1 into account. Currently, most of the assays employed cannot clearly distinguish between proliferation and migration and partly neglect the MACC1-induced effects on proliferation.

Despite the high effort put into elucidating MACC1-induced effects, several questions regarding its promigratory effects remain open. Downstream targets mediating the currently discovered effects need to be analyzed more precisely, as by now mostly the major signaling routes are identified. As MACC1 was shown to affect cortical tension and some of its downstream targets affect key components of the actin cortex, the question arises if and how MACC1 affects blebbing and thus migration in low adhesive environments. Another question is how 3D migration is altered in MACC1 dependence, under different amounts of confinement, in matrices of varying stiffness and composition. Furthermore, it also appears necessary to elucidate the MACC1-dependent effects on changes in the adhesive system, currently summarized under the broad term EMT, as they largely determine collective migration, a key factor in tumor invasion. This includes substrate dependence of cell–matrix adhesion formation, the strength of cell–cell and cell–matrix adhesions, and consequences on biomechanical properties and collective migration. Further experiments should clarify whether MACC1 additionally affects heterotypic cell–cell interactions with stromal cells and what the consequences are.

Currently, several drugs are under clinical trials which have at least a partial inhibitory effect on cell migration, potentially affecting metastasis formation (234). Given the clear association between MACC1 and poor survival and increased metastasis in so many tumor entities and its tight relation to cell migration and the cytoskeletal or adhesive system, it is likely that targeting MACC1 and analyzing its signal cascades will help to better understand the metastatic processes and develop precise tools to interact with tumor progression.

## Author contributions

TH, UH and FD contributed to the writing and review of the manuscript. All authors contributed to the article and approved the submitted version.
